# Evaluation of Cost-Effective Strategies for Rabies Post-Exposure Vaccination in Low-Income Countries

**DOI:** 10.1371/journal.pntd.0000982

**Published:** 2011-03-08

**Authors:** Katie Hampson, Sarah Cleaveland, Deborah Briggs

**Affiliations:** 1 Boyd Orr Centre for Population and Ecosystem Health, Institute for Biodiversity, Animal Health and Comparative Medicine, University of Glasgow, Glasgow, United Kingdom; 2 Global Alliance for Rabies Control, Manhattan, Kansas, United States of America; Swiss Tropical Institute, Switzerland

## Abstract

**Background:**

Prompt post-exposure prophylaxis (PEP) is essential in preventing the fatal onset of disease in persons exposed to rabies. Unfortunately, life-saving rabies vaccines and biologicals are often neither accessible nor affordable, particularly to the poorest sectors of society who are most at risk and upon whom the largest burden of rabies falls. Increasing accessibility, reducing costs and preventing delays in delivery of PEP should therefore be prioritized.

**Methodology/Principal Findings:**

We analyzed different PEP vaccination regimens and evaluated their relative costs and benefits to bite victims and healthcare providers. We found PEP vaccination to be an extremely cost-effective intervention (from $200 to less than $60/death averted). Switching from intramuscular (IM) administration of PEP to equally efficacious intradermal (ID) regimens was shown to result in significant savings in the volume of vaccine required to treat the same number of patients, which could mitigate vaccine shortages, and would dramatically reduce the costs of implementing PEP. We present financing mechanisms that would make PEP more affordable and accessible, could help subsidize the cost for those most in need, and could even support new and existing rabies control and prevention programs.

**Conclusions/Significance:**

We conclude that a universal switch to ID delivery would improve the affordability and accessibility of PEP for bite victims, leading to a likely reduction in human rabies deaths, as well as being economical for healthcare providers.

**Author Summary**

Rapid delivery of post-exposure vaccination is essential for preventing the fatal onset of rabies in persons bitten by rabid animals. But for communities most at risk of exposure to rabies (in resource poor countries where domestic dog rabies is still common), post-exposure vaccines are often not affordable and are only available in limited quantities. Several safe and effective regimens for delivery of these vaccines are recommended by WHO, but these are inconsistently implemented and there are no clear recommendations as to which is the best regimen for specific settings. We developed a framework for comparing the cost-effectiveness of different vaccination regimens, including existing approved regimens and new candidates subject to approval, in terms of costs per death averted. We demonstrate that post-exposure vaccination is an extremely cost-effective public health intervention and that changing delivery from intramuscular to intradermal vaccination has considerable benefits. Large savings in the volume of vaccine required to treat the same number of patients could potentially both mitigate vaccine shortages and reduce delays in delivery, enabling wider vaccine distribution, and thus improving the accessibility and affordability of these life-saving vaccines. We also present financing mechanisms that could help subsidize the cost for those most in need, and even support new and existing rabies control and prevention programs, without compromising existing healthcare budgets.

## Introduction

Rabies is invariably fatal once clinical signs appear but can be readily prevented after exposure with prompt and appropriate post-exposure prophylaxis (PEP) [1]. PEP is therefore the most critical life-saving intervention essential for the prevention of rabies in humans after exposure [2]. In reality, most of the estimated 7 million people exposed to rabies each year live in resource poor countries where life-saving rabies vaccines are not always available or easily affordable [3], [4], [5], [6]. This stark situation contributes to the fact that almost all of the estimated 55,000 annual human rabies deaths occur in Africa and Asia where the virus circulates endemically in domestic dog populations [7].

The WHO-recommended PEP protocol includes immediate wound washing, expeditious administration of rabies vaccine, and for severe categories of exposure, infiltration of purified rabies immunoglobulin (RIG) in and around the wound [8]. RIG is rarely administered in low-income countries because it is expensive (from USD$25 to over USD$200 depending on whether it is of equine or human origin)[7], [9] and in short supply (see the following examples: [3], [4], [5], [10]). Therefore, it is usually only post-exposure vaccination (without RIG) that is administered to protect a bite victim from succumbing to rabies [3]. Several factors affect the likelihood of promptly obtaining and completing PEP vaccination. Vaccine vials cost from USD$7–20 in most low-income countries and multiple vials are required per patient contingent upon the PEP regimen used. In some countries, governments provide vaccine free-of-charge or subsidize its cost, but budgets allocated for this are often insufficient, resulting in shortages or leaving only a few centres with a reliable supply. Alternatively, victims pay for vaccine, but charges are often prohibitive [3]. Costs of travel and or accommodation accumulate according to the number of clinic visits that a patient and, in many cases, an accompanying family member, makes to complete PEP. These considerable indirect costs [7] are affected by vaccine availability, and rise during shortages when patients and families are forced to travel further (often to multiple clinics), wasting time and money [3]. Delays caused by shortages also reduce compliance. All too often victims fail to promptly obtain or complete PEP, which in the worst cases results in rabies deaths [3], [4], [5].

Following WHO approval of intradermal (ID) administration of PEP vaccines [11], there has been significant discussion of the value of ID versus intramuscular (IM) delivery [2], [12], [13], [14], [15], [16], [17]. The main argument is that ID vaccination is more economical because smaller volumes of vaccine can be used to elicit an equivalent immune response (0.1 mL for each ID injection versus one 0.5 or 1 mL vial for each IM injection). The caveat of ID regimens is that vaccine remaining in partially used vials must be discarded within 6 to 8 hours to minimize risks of bacterial contamination (current vaccines do not contain preservatives) [2], [18], which may be perceived as waste. Moreover, vial sharing amongst patients may lead to practical difficulties in health provider budgeting. All WHO-recommended PEP regimens for WHO pre-qualified vaccines are safe, immunogenic, and efficacious. Thus policy should aim to prevent failures in PEP delivery by preventing vaccine shortages, reducing costs for victims and healthcare providers and promoting patient compliance to ensure PEP efficacy. The variety of WHO-recommended PEP regimens allows flexibility, but can lead to confusion regarding which regimen best meets the needs for a specific setting. Additionally, new regimens are continually being developed that require evaluation prior to implementation. Here, we provide a framework for comparing cost-effectiveness of different PEP regimens (including existing approved regimens and new candidates subject to approval) from the perspective of both healthcare providers and bite victims under a range of scenarios (from low to high throughput clinics) and under realistic constraints (poor patient compliance and some vaccine wastage). We discuss the implications for PEP affordability, availability and accessibility and offer recommendations for policy formulation and vaccine research.

## Methods

**Figure 1:**
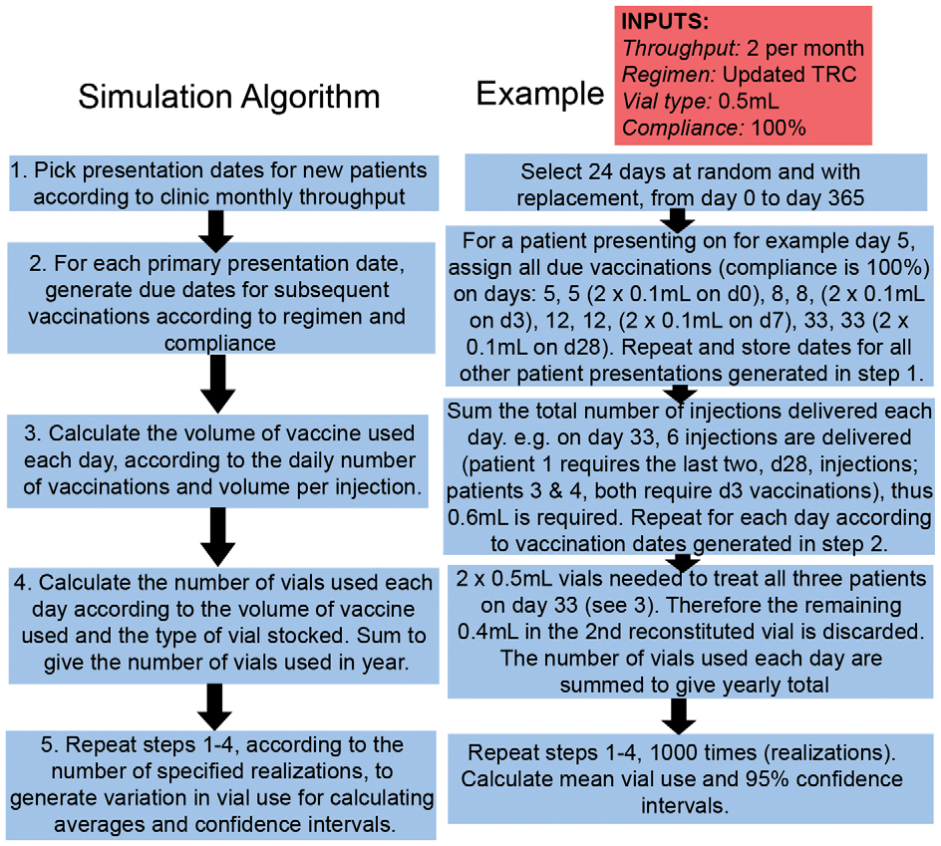
Simulation framework for determining vaccine use under different PEP regimens and model inputs. Framework for exploring different model inputs (detailed in Table 3) including degree of patient compliance, types of vaccine vials and levels of vaccine waste.

We developed a simulation framework for evaluating vaccine use under different PEP regimens. We compared different vaccination regimens (detailed in Table 1) that are currently approved by WHO and the Advisory Committee on Immunization Practices (ACIP), together with two additional candidate regimens (the 4-site and 1-week ID PEP regimens), that have recently undergone clinical trials and could potentially be used in future conditional upon review and approval by WHO. The algorithm for our simulations is shown in Figure 1 with an example scenario.

**Table 1:** Attributes of rabies post-exposure vaccination regimens.

Regimen Name	Clinic visits required	Schedule of visits (day)	Injections per visit	Vials opened/ accessed per PEP course	Volume of vaccine used for 1 course of PEP (mL)**	Route of administration	Approval status	References
Essen 5-dose	5	0,3,7,14,28	1,1,1,1,1	5	5 (2.5)*	IM	WHO 1992-	[11]
Essen 4-dose	4	0,3 7,14	1,1,1,1	4	4 (2)*	IM	ACIP 2009-	[21]
Zagreb	3	0,7,21	2,1,1	4	4 (2)*	IM	WHO 1992-	[34]
Thai Red Cross ID	5	0,3,7,28,90	2,2,2,1,1	5	0.8	ID	WHO 1992	[34]
Updated Thai Red Cross ID	4	0,3,7,28	2,2,2,2	4	0.8	ID	WHO 2005-	[32], [35]
4-site ID	4	0,7,28,90	4,2,1,1	4	0.8-0.9	ID	Not yet approved	[36]
1-week ID	3	0,3,7	4,4,4	3	1.2–1.5	ID	Not yet approved	[37]

We do not include the rarely used 8-site ID regimen [32] as it was recently recommended that this be removed from the list of WHO-approved ID regimens to simplify and facilitate the use of ID PEP [33]. We did not include the Thai Red Cross (TRC) regimen in analyses, because the updated TRC is always preferable requiring 4 rather than 5 clinic visits, thus entailing fewer indirect costs.

ID = intradermal, IM = intermuscular.

ACIP = Advisory Committee on Immunization Practices, USA.

*Calculated assuming 0.5 mL vials are used. **For ID regimens that use 0.4 mL or 0.8 mL of vaccine in a single hospital visit, we assume that in many cases a whole vial of vaccine will be divided between injection sites.

### Cost data

We used cost data associated with rabies reported from previous studies (Table 2). These include direct (medical) costs corresponding to rabies vaccines and their administration and indirect (non-medical) costs including transport to and from clinics and loss of income while receiving PEP. We assume that the time taken to vaccinate a patient is equivalent for both ID and IM administration and we did not include costs of RIG because most bite victims in Africa and Asia do not receive RIG [3], [4], [5].

**Table 2:** Costs associated with PEP vaccination.

Cost type	Parameter	Estimate (USD)	Reference
Medical	Material costs per injection (needles, syringes, swabs)	**$0.1**	[7], Global
		$0.4	[38], India
	Overhead costs per clinic visit (staff salaries, administration)	**$0.5**	[39], Global
		$1.2	[38], India
	Vaccine costs per vial	$8.75–11.5	[29], Thailand
		$7.5	http://www.msd.or.tz/ Tanzania
		$8–20	M. Sambo, pers com, Tanzania,
		$11	J. Girardi, pers com, Indonesia
		$18	[40], China
		$7–18	[41], South-East Asia
		**$10**	[7], WHO procurement services
		$7–9	D. Xuyen, pers com, Vietnam
		$6.6	[38], India
Non-medical	Transport, accommodation and income loss costs per clinic visit	**$2.9–5.5**	[7], Africa, Asia
		**$7–14**	[42], Tanzania

Costs vary within and between countries. For the purpose of comparison, in our simulations we used the values that are emboldened, but absolute cost-effectiveness will depend on specific settings. Note that for analyses of costs from the patient perspective, we explore the full range of indirect costs.

### Definitions

We explored vaccine use according to different model inputs that are detailed in Table 3 and defined as follows:

**Table 3:** Model inputs and sensitivity analyses conducted during simulations.

Model inputs	Description	Range
Clinic throughput	Average number of new animal-bite patients presenting at a clinic each month	1–1000 new patients/month
Regimen	Intramuscular or intradermal regimen used for all animal bite patients	Essen 4-dose (IM), Zagreb (IM)Updated-TRC (ID), 4-site (ID), 1-week (ID)
Vial type	Vial size stocked by clinic	0.5 mL, 1 mL
Compliance	Probability of completing each subsequent visit for PEP vaccination	100% (complete compliance), 50% (poor compliance). In analyses we assume that increased costs reduce compliance, and specifically that there is complete compliance when a full course of PEP costs bite-victims $10 or less, but for every $1 increase in cost, compliance is reduced by 0.05%.
Wastage	The number of 0.1 mL injections that can be obtained from a vial	All opened vials must be discarded at the end of the day. Wastage: 4 injections from a 0.5 mL vial, and 8 from a 1 mL vial. No wastage: 5 injections from a 0.5 mL vial, and 10 from a 1 mL vial
Vaccination efficacy	Effectiveness of each subsequent vaccination visit in preventing the onset of rabies	We explored hypothetical additive protective efficacy of each vaccination day on the cost-effectiveness of regimens (from 0–100%). E.g. If each vaccination day provides 90% protection, with the updated TRC regimen, 90% of victims will be protected on d0, 99% on d7 and 99.9% on d14 and 100% on d28, whereas using the 1-week regimen 100% would be protected on d7.

#### Clinic throughput

The number of bite patients presenting to a clinic for the first time in need of PEP. In contrast, the overall number of patients presenting to a clinic depends on the PEP regimen in use, its schedule requirements (Table 1) and the degree to which patients comply.

#### Vial size

Most rabies vaccines are sold in 0.5 mL or 1 mL vials, at equal cost, which affects the number of patients that can share the vial for ID vaccinations.

#### Vaccine wastage

Opened vials must be used within 6–8 hours and in practice there will always be some wastage of vaccine. For regimens that use almost a complete vial (4×0.1 mL injections from a 0.5 mL vial) during a clinic visit (4-site and 1-week ID, Table 1), practitioners may opt to divide a single 0.5 mL vial to provide the four injections or a 1 mL vial to provide eight injections. We therefore compared scenarios whereby different numbers of 0.1 mL injections can be obtained from a vial (according to vial size, see Table 3).

#### Patient compliance

The probability of a bite patient returning to a clinic for subsequent PEP vaccination(s). Poor compliance has consequences for vaccine use, vial sharing and PEP efficacy (see below). We investigate compliance in terms of the probability of returning for each visit rather than variability in the date of return. We assume patient compliance is affected by the cost of obtaining PEP and explore the implications of this relationship.

#### Vaccination efficacy

For the purposes of these comparative analyses, we assumed 100% efficacy of complete PEP vaccination in preventing rabies cases for all regimens. In the absence of PEP vaccination we assume that just under 20% of victims of bites by rabid animals develop rabies and that all clinical cases of human rabies result in death [19]. Incomplete PEP vaccination is less effective and almost 10% of human rabies cases reported from study in India had received incomplete PEP vaccination with CCVs [20]. However we were unable to find data on the impact of incomplete PEP vaccination on the likelihood of rabies onset. Rabies virus neutralization antibody titres increase following primary vaccination, generally peaking between days 14–28 [13], [21], [22]. Therefore for scenarios with less than 100% patient compliance we assume additive protection with each consecutive vaccination and explore a range of efficacies (Table 3).

We ran 1000 realisations (see Figure 1 example) for each scenario to capture variation in dates of patient presentation and consequences for vial sharing.

### Outcomes

We analyzed outcomes in terms of savings in vaccine use, human rabies cases averted and incremental cost-effectiveness ratios. We evaluated incremental cost-effectiveness ratios (ICER) in terms of dollars per rabies death averted: costs_PEP_/(Effectiveness_PEP_-Effectiveness_No PEP_), where subscripts refer to whether or not PEP vaccination was administered. We calculated cost-effectiveness from the perspective of the health provider and included only direct medical costs. We also modify this calculation of cost-effectiveness under poor compliance and according to hypothesized protective efficacy of incomplete vaccination (Table 3).

We compiled data on clinic throughputs in different settings and calculated the annual costs of PEP vaccination using different regimens for these settings. In practice, many clinics operate on a cost-recovery basis and charge for PEP. However, the number of new and returning animal-bite patients expected at a clinic on a daily basis cannot be precisely predicted, making it difficult to determine appropriate charges for ID administration. We compared four pricing strategies: 1) charging patients per injection according to the amount of vaccine used; 2) charging patients per injection at rates that are marginally higher than the price of the amount of vaccine used (illustrated with patients paying per injection at a rate that is 25% or 30% of vial costs); 3) charging patients a set price on their first visit (illustrated with a fee equivalent to 1.5 vials), but providing all subsequent doses without payment; 4) charging patients the price of one vial for each of their first and second hospital visits, but providing vaccine for free on subsequent visits. For all the strategies we assume that patients pay the costs of materials for vaccination and a consultation fee, which is equivalent to the price of overhead for a clinic visit in addition to the vaccination costs described above. We explored implications for cost recuperation (based on costs of PEP delivery shown in Table 2) by estimating annual savings of the different pricing strategies dependent upon throughput and the regimen in use. We present the net gains under these pricing strategies for the updated TRC regimen (the only currently WHO approved ID regimen) for clinics for which throughput data was compiled.

We compare the costs of PEP for bite-victims, depending upon the pricing strategies described above and including the provision of PEP free-of-charge and under different assumptions about indirect costs (based on the range of indirect costs in Table 2). Specifically we assume that bite victims travel further to reach a clinic in rural rather than urban settings and incur correspondingly higher costs. We also calculate the likely risks for patients of poor PEP compliance according to assumptions about vaccine efficacy (Table 2) and explore how costs may affect compliance with PEP regimens and implications for the risk of developing rabies.

All analyses were performed using the statistical programming language R. Scripts implementing our simulations are available upon request.

## Results

**Figure 2:**
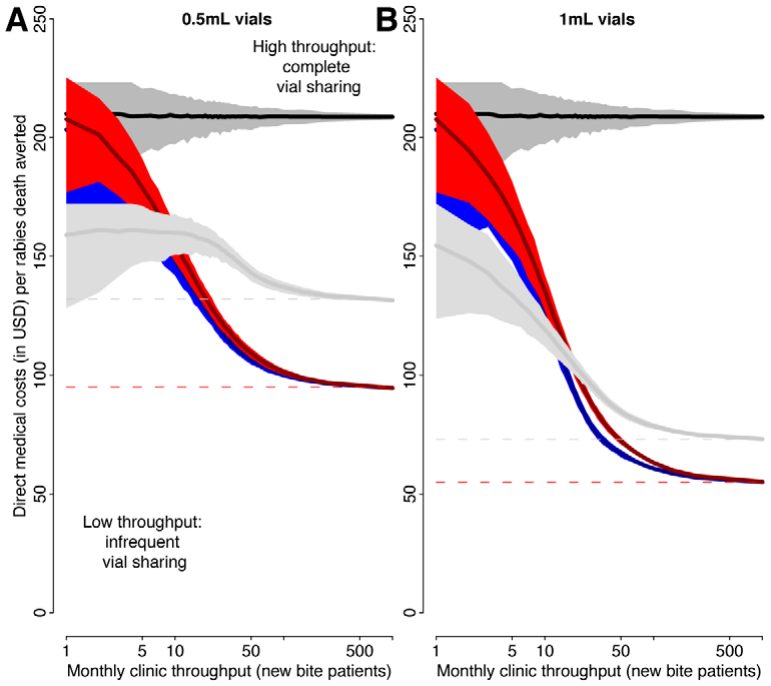
Cost of vaccination per rabies death averted for different PEP regimens according to clinic throughput. Costs for IM administered vaccinations (the Zagreb regimen and the Essen 4-dose reduced regimen are exactly equivalent and shown in black) and ID administered vaccinations (the updated TRC regimen is shown in blue, the 4-site in red, and the 1-week in grey) per rabies death averted is plotted against clinic throughput (the number of new animal bite patients presenting for PEP vaccination each month). Shading represents 99% confidence intervals resulting from variation in patient arrival dates and the effects on vial sharing. Dashed lines highlight optimal vaccine use in high throughput clinics. Panel A is based on 0.5 mL vials and panel B on 1 mL vials. Here, we assume that vaccine is perfectly delivered without any wastage (5 complete 0.1 mL injections from a 0.5 mL vial, and 10 complete 0.1 mL injections from a 1 mL vial), but we show the reductions in efficiency assuming some wastage in Figure S1. Note the x-axis is plotted on a log scale.

For IM vaccination, both the reduced 4-dose Essen and the Zagreb regimens are more economical than the 5-dose Essen because they use only 4 vaccine vials in comparison to 5 vials for the 5-dose schedule, i.e. 80% of the total volume of vaccine (Table 1). All ID regimens use less vaccine than IM regimens and are cost less per rabies death averted (Figure 2). Clinic throughput generally increases the cost-effectiveness of ID vaccination, with high throughput clinics most cost-effective and low throughput clinics least cost-effective. The only exception to this is the situation where some wastage is assumed and 0.5 mL vials are used, in which case the 1-week ID regimen does not increase in cost-effectiveness with throughput (Figure S1), but is still considerably more cost-effective than IM regimens.

The updated TRC and the 4-site ID regimens were the most cost-effective in high throughput settings (between $100 and $55 per life saved depending on throughput and vial size). While in very low throughput clinics, the 1-week ID regimen was the most cost-effective ($160–150 per life saved, Figure 1). In high throughput clinics the updated TRC and 4-site ID regimens use just 40% of the volume of vaccine in comparison to preferred IM regimens (Essen 4-dose and Zagreb) when 0.5 mL vials are used and 20% of the volume when 1 mL vials are used (Table 1, Figure 1). The cost-effectiveness of all ID regimens increases considerably when 1 mL vials are used instead of 0.5 mL vials (Figure 1). The estimated costs of PEP vaccination to health providers are shown for different regimens in Table 4 for a variety of throughput settings and illustrate how provision of PEP using ID regimens is considerably more economic than provision of PEP using IM regimens.

**Table 4:** Annual estimated costs of PEP vaccination per clinic and savings from different pricing strategies.

			Regimen costs (USD)					Expected annual savings (USD):			
								$2.5/injection*	$3/injection	$15/course	
Setting	Route	Monthly throughput	Essen 4-dose	Zagreb	updated TRC	4-site ID	1-week ID	1 mL vials	1 mL vials	1 mL vials	Reference
Tanzania (rural – urban)	IM, ID**	∼15–400	7,150–190,350	7,100–188,500	3,700–60,800	4,100–61,500	3,800–80,850	400–48,650	1,150–67,900	−500–24,650	[3], M. Sambo & M. Kiboko, pers comm.
Bali (rural – urban)	IM	600–9000	285,500–4,282,400	282,700–4,240,750	91,200–1,330,850	92,200–1,334,150	121,300–1,795,800	73,000–1,131,600	101,800–1,563,600	37,000–591,550	[31]
Chad (urban, N'djamena)	IM	∼30	14,300	14,150	5,750	6,350	6,800	2,500	3,900	660	[28]
Uganda (across country)	IM	∼17	8,100	8,000	3,950	4,400	4,200	700	1,500	−300	[43]
Manila, Philippines	ID	>600	>285,500	>282,700	>91,200	>92,200	>121,300	>73,000	>101800	>37,000	[2]
Phnom Penn, Cambodia	IM	>1200	>571,000	>565,450	>177,450	>177,900	>239,400	>150,900	>208,450	>78900	[4]
India	IM	>4000	>1,903,300	>1,884,800	>591,500	>592,950	>798,100	>502,900	>694,900	>262,900	G. Sampath, pers comm.

**Figure 3:**
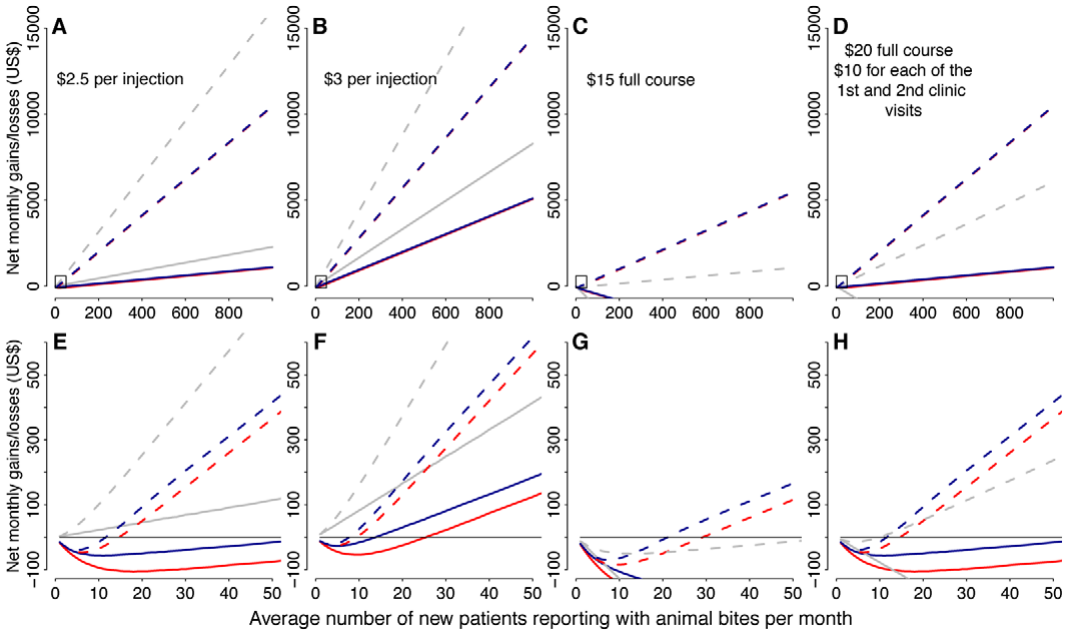
Clinic monthly savings and losses from ID administration of PEP under different pricing mechanisms. A & E) patients are charged $2.5 per injection (25% of vial costs assuming a single vial costs $10) as well as a consultation fee and materials costs (see Table 4 for the costs from the perspective of the bite victim). B & F) patients are charged $3 per injection (30% of vial costs) as well as a consultation fee and materials costs. C & G) patients are charged a flat rate of $15 for the full PEP course (the cost of 1.5 vials) as well as a consultation fee and materials costs. D & H) patients are charged a flat rate of $20 for the full PEP course ($10 for each of the first two clinic visits, equivalent to two vials) as well as a consultation fee and materials costs. A, B, C, & D) compare regimens across a range of patient throughputs (from very low to very high, 1–1000 patients per month). E, F, G & H) are a closer examination of costs in low throughput clinics (1–50 patients per month). Blue lines indicate the updated TRC ID, red lines indicate the 4-site ID and gray lines indicate the 1-week ID regimens respectively, with solid lines corresponding to 0.5 mL vials and dashed lines corresponding to 1 mL vials. The pricing strategies shown would all result in substantial losses for IM delivered PEP vaccination (not shown).

Savings are calculated from recovered costs from charging for the updated TRC PEP regimen according to the strategies described (see methods for full details) minus costs of PEP delivery (estimated from costs in Table 2) assuming use of 1 mL vials. For all these scenarios we assume there is some wastage and therefore 8×0.1 mL injections are obtained from each 1 mL vial. Costs increase and savings decrease when 0.5 mL vials are used, as illustrated in Figure 3.

*: For the updated TRC regimen, $2.5 per injection is equivalent to $20 for the full course.

**: In parts of Tanzania the updated TRC ID regimen has been proposed for implementation.

When health providers charge for PEP vaccination according to the strategies described, substantial costs are recovered when using ID regimens and in most cases savings are made. The extent of savings and how these vary with clinic throughput for different ID regimens are shown in Figure 3. Using 1 mL vials rather than 0.5 mL vials increases savings for all pricing strategies. Charging patients for exactly the amount of vaccine administered using the ID route results in a net loss for healthcare providers except in high throughput clinics. Charging patients per ID injection at rates slightly greater than the price of the vaccine used results in net savings in most locations (Figure 3 shows savings for charging $2.5 or $3/injection assuming vials cost $10). Other strategies such as charging higher rates but for the primary presentation only, or for primary and secondary presentations only, also result in significant savings for high throughput clinics and losses occur only in very low throughput clinics when 1 mL vials are used. For example, when using 1 mL vials, charging the price of a vial for each of the first 2 presentations, results in savings in clinics that receive over 15 new patients per month with all the ID regimens, whereas losses are always incurred when using 0.5 mL vials in lower throughput clinics (Figure 3). Similar savings are made in high throughput clinics charging a fixed price for a full PEP course when 1 mL vials are used (illustrated by a $15 set rate, assuming a vial costs $10, in Figure 3), however, charging $15 for a full ID course (all regimens) is not sufficient to recuperate costs when using 0.5 mL vials. Extrapolations assuming use of the $15 full course of the updated TRC regimen with 1 mL vials (the only ID regimen currently recommended by WHO) suggest that even in countries with mainly low throughput clinics (e.g. Tanzania), savings recuperated from urban centres would ensure sustainability, and in the highest throughput settings annual savings could exceed $100,000 in a single clinic (Table 4).

Where PEP vaccination is provided free-of-charge, the Zagreb IM and the recently proposed 1-week ID regimens are most preferable for patients, who incur only indirect costs (Table 5). This is because only 3 hospital visits are required as compared to the Essen IM and the updated TRC and 4-site ID regimens, which all require 4 visits (Table 1). When patients are required to pay for PEP vaccination, the most preferable regimen for bite victims varies depending on pricing strategies and relative travel costs (Table 5). However, in terms of price, ID regimens are always preferable over IM regimens. When travel costs are low and PEP is charged per injection, the updated TRC and the 4-site ID regimens are preferable. The 1-week ID regimen is preferable when travel costs are high, and particularly when flat rates are charged for the full PEP course, rather than per injection (Table 5).

**Table 5:** Costs of PEP vaccination regimens from the bite-victim perspective.

	Travel costs only (USD$):		$2.5 per injection:		$3 per injection:		$15 full course:		$10 for 1st and 2nd visits:	
Regimen	Near^1^	Far^2^	Near^1^	Far^2^	Near^1^	Far^2^	Near^1^	Far^2^	Near^1^	Far^2^
Updated TRC ID	11.6	56	**34.4**	78.8	**38.4**	82.8	29.4**	73.8**	34.4	78.8
4-site ID	11.6	56	**34.4**	78.8	**38.4**	82.8	29.4**	73.8**	34.4	78.8
1-week ID	**8.7**	**42**	41.4	**74.7**	47.4	**80.7**	**26.4** **	**59.7** **	**31.4** **	**64.7** **
Essen 4-dose*	11.6	56	54*	98.4*	54*	98.4*	54*	98.4*	54*	98.4*
Zagreb*	**8.7**	**42**	50.6*	83.9*	50.6*	83.9*	50.6*	83.9*	50.6*	83.9*

Costs were calculated based on whether PEP vaccination is provided free-of-charge, or according to different pricing strategies. The most affordable regimens are emboldened for each strategy. We assume that for each clinic visit patients pay a consultation fee (that is equivalent to the price of overhead for a clinic visit) and the costs of materials for injections (Table 2).

1 Low indirect costs are assumed to be $2.9/visit (best case scenario in Table 2), corresponding to patients from urban areas that only need to travel relatively short distances to obtain PEP.

2 High indirect costs are assumed to be $14/visit (worst case scenario in Table 2), which corresponds to patients from rural areas, that have to travel long-distances to a hospital and may need to stay overnight whilst seeking PEP.

*: For IM regimens, when patients pay for PEP vaccination we assume they pay $10/vial (Table 2).

**: When using 0.5 mL vials, charging $15 for a full course does not recuperate costs for any ID regimen and charging $10 for each of the first two clinic visits does not recuperate costs for the 1-week ID regimen (see Figure 3).

**Figure 4:**
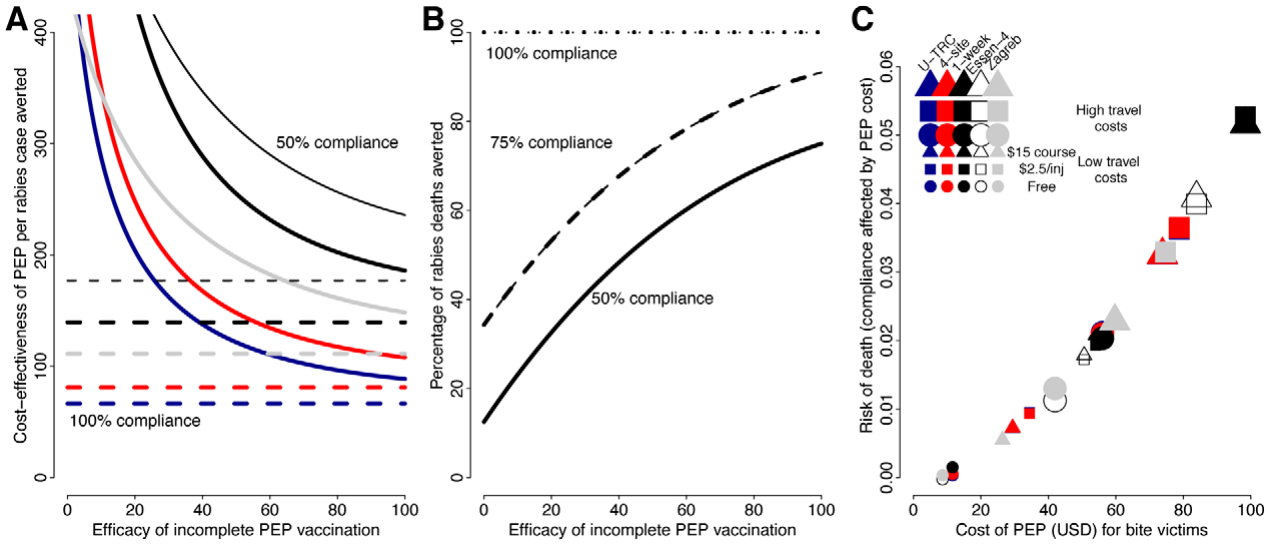
Effects of compliance on PEP effectiveness in preventing rabies and cost-effectiveness per death averted. A) Additive protective efficacy of PEP (defined in Table 3) given 50% compliance is plotted against cost-effectiveness per rabies death averted, calculated from direct medical costs (Table 2). The different regimens are indicated as follows: reduced 4-dose Essen IM regimen in thick black, Zabreb IM in thin black, updated TRC ID in blue, 4-site ID in red and 1 week ID in gray. The dashed lines correspond to complete compliance. Here we assume use of 0.5 mL vials and clinic throughput of 100 new bite patients per month. Assuming use of 1 mL vials results are qualitatively similar but more cost-effective. B) Additive protective efficacy of PEP regimens (Table 3) is plotted against the percentage of rabies deaths averted at high (100%, dotted lines), moderate (75%, dashed lines) and poor (50%, solid lines) levels of compliance. Thick lines correspond to regimens requiring 4 clinic visits (reduced Essen 4-dose IM, updated TRC ID) and thin lines correspond to regimens requiring 3 clinic visits (Zagreb IM, 4-site ID, 1-week ID). C) The costs of PEP regimens for bite victims according to different pricing strategies (Table 5) are plotted against the risk of developing rabies assuming 75% additive protective efficacy for each PEP visit and assuming that patient compliance is affected by PEP costs (as described in Table 3).

We assume that high costs reduce patient compliance, which in turn reduces the effectiveness of PEP in preventing rabies and thus the cost-effectiveness of PEP. Specifically, we assume 100% compliance when patients pay $10 or less for PEP, and that for every dollar increase there is a 0.05% reduction in compliance (Table 3), thus the most expensive regimen ($98.4 per course for the Essen 4-dose IM for patients with high travel costs, Table 5), has only 42.8% compliance. The cost per rabies death averted decreases as the efficacy of the regimen increases, and therefore cost-effectiveness increases and a greater proportion of preventable deaths are averted (Figure 4A & B). Cost-effectiveness is lowest at low levels of compliance. The proportion of deaths prevented also increases with vaccine efficacy (Figure 4B). At low levels of vaccine efficacy, regimens that require 3 clinic visits (Zagreb, 4-site ID, 1 week ID) prevent a greater percentage of deaths than regimens that require 4 clinic visits (Essen 4-dose, updated TRC). Overall, the risk of death increases with the costs of PEP as patients become less likely to comply with regimens (Figure 4C). For all pricing strategies that we present, patients who live further from clinics have reduced compliance and heightened risks. When PEP vaccination is free of charge, risks are minimized, and risks are maximized when charging for IM regimens (Figure 4C).

## Discussion

Rabies post-exposure vaccination is essential for preventing this fatal disease but can be out of the financial reach of many bite victims. Vaccine shortages are common in developing countries and due to limited availability bite victims often need to travel long distances to obtain vaccine. Thus, patients often incur substantial costs and face dangerous delays in securing PEP and avoidable human rabies deaths occur as a direct result of poor access to affordable PEP [3], [4], [5]. We examined the costs of IM versus ID administration of PEP vaccine in different settings and under realistic constraints such as poor compliance. We demonstrate that ID delivery of PEP is considerably more cost-effective than IM delivery in terms of averting rabies cases and saving lives. Clinic throughput affects the capacity for vial sharing, and therefore the cost-effectiveness of ID administration relative to IM. As throughput increases, ID regimens become increasingly cost-effective, using up to 80% less vaccine (Figure 1). Yet, even clinics with relatively low throughput (∼10 new patients/month) would reduce vial use by 25% by switching from IM to ID administration of PEP. Increased use of ID regimens could therefore prevent vaccine shortages and enable wider vaccine distribution, both increasing the number of patients that can be treated and the overall accessibility of PEP. Concurrent changes in PEP costs to patients could also improve affordability, while providing incentives for compliance without compromising existing health budgets. These issues should be considered in the design of PEP policy because they could reduce the burden of rabies by increasing the availability of vaccines for the rural poor who bear the brunt of rabies in most developing countries.

Our principal finding that ID administration of PEP is more cost-effective than IM administration and reduces the amount of vaccine used is important given the frequency with which PEP vaccine shortages occur at clinics in many developing countries. Savings in vaccine use are substantially larger when using equivalently priced 1 mL rather than 0.5 mL vials, especially in high throughput clinics because of greater vial sharing. In this situation there is no advantage to stocking a mixture of vials (100% of 1 mL vials is always most cost-effective), but should pricing change (so that 1 mL and 0.5 mL vials differ in price), optimal stocking strategies should be evaluated as a priority. For safety reasons (potential for contamination) vial sharing is only possible on the day of vaccine reconstitution, even though potency remains high when properly stored [23]. Research into methods of preserving rabies vaccines and preventing contamination could therefore enable more economical use of vaccines, including production in larger volume vials.

Despite policies to provide PEP free-of-charge, many bite victims need to pay to promptly obtain PEP. In the light of this, a switch to ID administration could reduce costs to bite victims. But, there are many ways to charge for PEP. Only in high throughput locations, where vials can be shared completely, could patients be charged exactly for vaccine used without clinics operating at a loss. Rates could be set proportional to clinic throughput to prevent losses and ensure cost-recovery, but this would result in inequities (with higher throughput clinics providing cheaper PEP) that would disadvantage patients attending lower throughput clinics, i.e. the rural poor. More equitably, patients could be charged set rates that are much lower than for IM PEP (Table 5), whilst ensuring cost recovery (Figure 3). Savings (see Table 4) from higher throughput clinics could subsidize either lower throughput clinics that might operate at a loss, or the poorest patients who are unable to afford PEP (e.g. as part of a rolling fund or an insurance system) or even other rabies control and prevention activities. Innovative financing mechanisms could provide more affordable PEP and generate potentially high returns from high throughput clinics, but effective monitoring would be critical.

Health policy aims to reduce the burden of disease, but conflicts inevitably arise between the individual interests of patients and the population-level interests of healthcare providers. Choices about which regimens are preferable depend upon whether indirect or direct costs are a greater obstacle to bite victims. When PEP is provided free-of-charge, the recently developed 1-week ID regimen and the Zagreb IM are most advantageous for patients, because they entail fewer clinic visits. But both have drawbacks as the 1-week ID regimen is not yet approved by WHO and the Zagreb regimen, which is approved by WHO, uses more vaccine. If budgets and therefore vaccine supply are limited, the 4-site, if eventually approved by WHO, and updated TRC ID regimens are most preferable. Policies need to balance these issues to reduce costs for bite victims and prevent shortages.

A further consideration in PEP delivery is how to promote compliance and therefore improve the effectiveness of PEP. We assume that affordability of PEP will improve compliance and provision of PEP free-of-charge is therefore the ideal solution (Figure 4). However, when charging for PEP, flat rates that are more affordable than IM regimes (e.g. $15 for a full course or $20 for the first two visits, see Table 2) might incentivise compliance, but would recuperate costs only when 1 mL vials are used. Alternatively wider distribution of vaccine (even when charging for PEP) could reduce indirect costs for bite-victims and improve compliance. Staying in the vicinity of a clinic rather than travelling back and forth for each scheduled vaccination might also be cheaper for bite-victims, which would apply particularly for the 1-week ID regimen (we do not currently explore such complexities but data could inform model inputs for future analyses). Although the 1-week ID uses more vaccine than other ID regimens, reduced indirect costs could make it more affordable for bite-victims. This may facilitate compliance and has the added benefit of earlier complete protection reducing anxiety for bite-victims. In contrast, for the 4-dose ID regimen, the last dose of vaccine is not administered until day 90, which could reduce compliance in comparison to other ID regimens. Further study is therefore warranted to better quantify indirect costs of obtaining PEP and to understand the major constraints to PEP access and compliance for those most in need. Incomplete and late PEP is less effective in preventing the onset of disease, but there are no data available to quantitatively compare risks to full compliance (we were only able to explore hypothetical changes in PEP effectiveness with poor compliance). Contact tracing could potentially reveal more about these issues, and longitudinal serology studies could provide a useful proxy measure for immunogenicity that could be used to inform PEP policy.

Despite being more economical, misperceptions about ID, the lack of strong recommendations and a profusion of complex schedules have deterred their widespread adoption. Yet our analyses show that switching from IM to ID administered PEP has benefits to patients and healthcare providers. The updated TRC is the only currently WHO approved ID regimen, but the 4-site ID regimen is also highly cost-effective and the 1-week ID has other benefits for bite-victims. Their further evaluation by WHO is clearly warranted. More generally, since ID procedures involve delivery of only small amounts of vaccine, in order to apply our findings to settings where non pre-qualified vaccines are used, rigorous evaluation of the product including manufacturing standards, safety, immunogenicity and efficacy must be prioritized.

The absolute cost-effectiveness of PEP depends upon the regimen, clinic throughput, clinic overhead and costs of materials for vaccine delivery and vaccine vials. Nonetheless, our estimates suggest that PEP is more cost-effective in averting deaths than childhood immunization through the Expanded Program on Immunization (USD$205/death averted in sub-Saharan Africa and South Asia [24]), which is considered one of the most cost-effective health interventions available [25]. Even considering vaccine waste, the worst-case scenario for PEP cost-effectiveness is around $200/death averted (for IM regimens) and in high throughput clinics use of ID regimens can reduce costs to just $60/death averted. Cost-effectiveness will decline if PEP is administered to patients who are bitten by non-rabid animals. We do not currently factor this into our calculations, but positive predictive values obtained from field data in Cambodia and in Tanzania [4], [26] suggest that PEP is largely administered to genuine rabid bite victims and that cost-effectiveness will remain high even with liberal provision of PEP [19]. Thus effective PEP delivery should be considered an extremely cost-effective investment for public health, given the current poor availability of this life saving intervention. However, rabies can only be eliminated through intervention in the animal reservoir [27], and this is likely to be the most cost-effective way of averting human rabies deaths in the long-term [28].

Our model has several simplifications, which could be elaborated on in future. We assume that the day of the week does not affect the likelihood of presenting for PEP vaccination. But patients may be less likely to present on Sundays (in many countries clinics providing PEP are not open on Sundays) and/or more likely to present on Mondays or other days of the week (e.g. after pay day), which may affect vial sharing. We also do not include pre-exposure vaccination. Livestock officers and extension workers involved in animal vaccinations and more at risk of animal bites should be pre-vaccinated. Pre-exposure vaccinations would likely make PEP more cost-effective (as less vaccine is required in the event of an exposure) and preliminary vaccinations could be coordinated to ensure effective vial sharing (e.g. prior to dog vaccination campaigns). But, in general in low-income countries, such pre-vaccinated persons are rare relative to non-vaccinated bite victims. In some high-risk settings pre-vaccination of children is under consideration [29], and our simulation framework could be useful for their further evaluation.

The availability and affordability of PEP is critical in determining the burden of rabies. Incidence in resource poor countries is directly affected by the inability of bite victims to obtain PEP and obtain it promptly. Reducing the cost of PEP and preventing administration delays is therefore particularly important in resource-limited settings. The variety of PEP regimens, vial sizes, and routes of administration has also made the delivery of these life-saving vaccines unnecessarily complicated. Our results provide evidence to show that a simplification to universal ID delivery of PEP could have massive advantages in low-income countries: streamlining guidelines, reducing the volume of vaccine use, mitigating vaccine shortages and making PEP more affordable to the most vulnerable. Health workers routinely deliver childhood immunizations intradermally, so there should be no technical difficulty in switching to ID administration. ID vaccination is as safe and efficacious as IM vaccination and is well-tolerated [30]. The immense advantages of ID PEP delivery should be specifically highlighted in outbreak situations, such as those recently reported from Bali [31] and in areas where vaccine supply is limited, as considerably more bite victims can be protected using the same volume of vaccine.

## Supporting Information

Figure S1 Cost of vaccination per rabies death averted (life saved) for different PEP regimens according to clinic throughput and assuming imperfect vaccine use (4×0.1 mL injections from a 0.5 mL vial, and 8×0.1 mL injections from a 1 mL vial). Costs for IM administered vaccinations (the Zagreb regimen and the Essen 4-dose reduced regimen are exactly equivalent and shown in black) and ID administered vaccinations (the updated TRC regimen is shown in blue, the 4-site in red, and the 1-week in grey) per rabies death averted is plotted against clinic throughput (the number of new animal bite patients presenting for PEP vaccination each month). Shading represents 99% confidence intervals resulting from variation in patient arrival dates and the effects on vial sharing. Dashed lines highlight optimal vaccine use in high throughput clinics. Panel A is based on 0.5 mL vials and panel B on 1 mL vials. Note the x-axis is plotted on a log scale.
(0.03 MB PS)
